# Trends in hypertension and hypertension treatment in primary care in general practices in Germany between 2013 and 2022

**DOI:** 10.3389/fcvm.2024.1390902

**Published:** 2024-06-12

**Authors:** Karel Kostev, Sarah Krieg, Louis Jacob

**Affiliations:** ^1^ Epidemiology, IQVIA, Frankfurt, Germany; ^2^ Department of Inclusive Medicine, University Hospital Ostwestfalen-Lippe, Bielefeld University, Bielefeld, Germany; ^3^ Research and Development Unit, Parc Sanitari Sant Joan de Déu, CIBERSAM, ISCIII, Dr. Antoni Pujadas, Barcelona, Spain; ^4^ AP-HP, Université Paris Cité, Lariboisière-Fernand Widal Hospital, Department of Physical Medicine and Rehabilitation, Paris, France; ^5^ Université Paris Cité, Inserm U1153, Epidemiology of Ageing and Neurodegenerative Diseases (EpiAgeing), Paris, France

**Keywords:** trends, hypertension, treatment, general practices, Germany

## Abstract

**Background:**

There is a scarcity of data on the epidemiology of hypertension and its treatment in Germany in recent years.

**Objectives:**

The present study aimed to investigate trends in the number of adults diagnosed with hypertension and those being prescribed antihypertensive drugs each year in general practices from this country between 2013 and 2022.

**Methods:**

This retrospective cohort study used data of adults aged ≥18 years continuously collected from 336 general practices in Germany (IQVIA) during 2013–2022. The diagnosis of hypertension and the prescription of antihypertensive drugs were coded using the ICD-10 and the EphMRA classification, respectively. Covariates included the total number of patients, mean (SD) age of patients, and proportion of women per practice. Trends in hypertension diagnosis and treatment were studied using linear regression models.

**Results:**

The mean (SD) total number of patients per practice ranged from 2,235 (1,055) in 2013–2,845 (2,090) in 2021 (*p*-value < 0.001). The mean (SD) age of patients per practice was between 48.4 (21.5) and 50.5 (21.4) years, while the proportion of women was 52.1%–53.9% (*p*-values < 0.001). After adjusting for covariates, there was a significant decrease in the number of patients diagnosed with hypertension (beta coefficient = −7.91, *p*-value < 0.001) and treated with any antihypertensive drug per practice per year between 2013 and 2022 (beta coefficient = −5.40, *p*-value < 0.001).

**Conclusion:**

This study identified decreasing trends in the diagnosis and treatment of hypertension in general practices in Germany in the last decade. These data may suggest that the prevention of hypertension has improved in this country in recent years.

## 1 Introduction

Hypertension, also known as high blood pressure, is a chronic condition defined as systolic blood pressure higher or equal to 140 mmHg or diastolic blood pressure higher or equal to 90 mmHg (1). The number of hypertension-related deaths per 100,000 people increased from 97.9 in 1990–106.3 in 2015 (2). Compared with the general population, individuals with hypertension are at an increased risk for premature mortality (3) and are more likely to be diagnosed with physical diseases [e.g., myocardial infarction (4) and stroke (5)] and have lower quality of life (6, 7). Furthermore, hypertension is associated with a major economic burden, with high direct and indirect costs (8). In this context, public health measures should aim at the prevention of hypertension in primary care, and to be effective, these measures should rely on recent epidemiological data.

In the last decade, several studies have focused on the trends of the prevalence of hypertension (9–18). There is some discrepancy in the findings of this literature, and trends have been found to increase (9, 15, 16, 18), stagnate (10, 11, 14), or decrease (12, 13, 17). Although this body of research has advanced the field, there are several limitations that should be acknowledged. First, a substantial proportion of these studies were conducted in China (15, 16) and the United States of America (USA) (11, 12, 18), and their results may not be extrapolated to other countries. Second, in these studies, trends in the prevalence of hypertension were analyzed prior to 2019, and there is no data on how the coronavirus disease 2019 (COVID-19) pandemic has impacted these trends. This lack of data is of concern, as some evidence suggests that COVID-19 may favor the onset of hypertension (19, 20) and that the related health crisis may have had deleterious effects on the management of hypertension (21). Third, to the best of the authors' knowledge, only one research has studied trends in the prescription of different antihypertensive drugs (e.g., angiotensin-converting enzyme inhibitors [ACEis], beta-blockers [BBs], and diuretics) (18). Taking these limitations into consideration, more data are warranted on how the diagnosis and treatment of hypertension have evolved in recent years.

Therefore, the goal of this longitudinal study was to investigate trends in hypertension (i.e., prevalence of diagnosis) and hypertension treatment (i.e., prevalence of treatment) in adults followed in general practices in Germany between 2013 and 2022. The study results may help in the implementation of public health measures preventing the occurrence of hypertension in this country.

## 2 Materials and methods

### 2.1 Database

The present study used data from the Disease Analyzer database (IQVIA). This database has already been described extensively in the literature (22). Briefly, the Disease Analyzer database includes data from general and specialized practices in Germany. The data are collected from the computers of the practices and anonymized before being sent to IQVIA every month. Data include demographics, diagnoses, and prescriptions. Diagnoses are coded using the International Classification of Diseases, 10th revision (ICD-10), while prescriptions are coded using the Anatomical Classification of Pharmaceutical Products of the European Pharmaceutical Market Research Association (EphMRA). Data quality is assessed by IQVIA on a regular basis, and this assessment relies on several criteria (e.g., completeness of data and linkage between diagnoses and prescriptions). Several factors are taken into account for the selection of practices to include in the database (e.g., age of practitioner, type of practice, community size, and German federal state). Finally, the Disease Analyzer database has been found to be representative of primary care practices in Germany and includes approximately 3% of all practices.

### 2.2 Study population, outcomes, and covariates

This study included all patients aged ≥18 years who were followed in 336 general practices in Germany between 2013 and 2022. General practices included in the analyses were those continuously sending data to IQVIA between 2013 and 2022, with a total number of 1,284 practices for this period. Individuals may have been followed for a single year or several years. The first outcome of the study was the number of patients diagnosed with essential hypertension (ICD-10 code: I10; incident and prevalent cases) each year between 2013 and 2022. The second outcome of the study was the number of patients being prescribed any antihypertensive drug, ACEis (EphMRA classification codes: C09A and C09B), angiotensin receptor blockers (EphMRA classification codes: C09C and C09D), BBs (EphMRA classification code: C07), calcium channel blockers (EphMRA classification code: C08), and diuretics (EphMRA classification code: C03).

### 2.3 Statistical analyses

The characteristics of the practices (i.e., the total number of patients, mean [standard deviation] age [in years], proportion of patients aged 18–39, 40–59, and ≥60 years, and proportion of women and men) were studied for each year between 2013 and 2022. These characteristics were compared between the 10 different years using the Kruskal-Wallis test for the total number of patients and the mean age of patients per practice and the Chi-square test for the other variables. The number of patients diagnosed with hypertension was described for each year using the mean (standard deviation) in the overall population and by age (i.e., 18–39, 30–59, and ≥60 years) and sex (i.e., female and male). Trends in the number of patients diagnosed with hypertension were further investigated using unadjusted and adjusted linear regression models with years included as a continuous independent variable and the number of patients diagnosed with hypertension as a continuous dependent variable. The adjusted model included as covariates the total number of patients, the mean age, and the proportion of women per practice for a given year. Finally, the number of adults being prescribed antihypertensive drugs (i.e., any drug, ACEis, ARBs, BBs, CCBs, and diuretics) was described for each year using the mean (standard deviation) in the overall population. Trends in the number of individuals receiving antihypertensive treatments were studied in the overall sample using unadjusted and adjusted linear regression models. In the adjusted linear regression model, the total number of patients, mean age, and proportion of women were included as covariates. Given that the COVID-19 pandemic may have impacted trends in hypertension diagnosis and treatment, analyses were repeated after excluding data collected after 2019. *p*-values lower than 0.050 were considered statistically significant. All analyses were performed using SAS version 9.4 (SAS Institute, Cary, North Carolina, USA).

## 3 Results

### 3.1 Characteristics of the general practices

The yearly characteristics of the 336 general practices between 2013 and 2022 are displayed in Table 1. The mean (standard deviation) number of patients per practice ranged from 2,235 (1,055) in 2013–2,845 (2,090) in 2021 (*p*-value < 0.001). In terms of age, the mean (standard deviation) age of patients per practice was at the lowest in 2013 [i.e., 48.4 (21.5) years] and the highest in 2020 [i.e., 50.5 (21.4) years], with the proportion of people aged ≥60 years ranging from 36.0% to 38.5% during the 10-year period. Regarding sex, the proportion of female patients per practice was between 52.1% in 2021 and 53.9% in 2014.

**Table 1:** Yearly demographic characteristics of the 336 general practices from Germany included in the study (2013–2022).

Variable	2013	2014	2015	2016	2017	2018	2019	2020	2021	2022	*p*-value
Mean (standard deviation) total number of patients per practice	2,235 (1,055)	2,238 (1,115)	2,306 (1,188)	2,338 (1,218)	2,351 (1,250)	2,382 (1,284)	2,369 (1,269)	2,374 (1,344)	2,845 (2,090)	2,680 (1,803)	<0.001
Mean (standard deviation) age per practice (in years)	48.4 (21.5)	48.8 (21.5)	49.0 (21.5)	49.0 (21.6)	49.5 (21.6)	49.7 (21.6)	50.0 (21.6)	50.5 (21.4)	50.1 (21.0)	49.9 (21.5)	<0.001
Proportion of patients aged 18–39 years per practice (%)	29.8	30.0	30.2	30.7	30.3	30.4	30.3	29.3	29.9	30.3	<0.001
Proportion of patients aged 40–59 years per practice (%)	34.2	33.6	33.4	32.9	32.6	32.4	32.0	32.2	32.9	31.9	
Proportion of patients aged ≥60 years per practice	36.0	36.4	36.4	36.4	37.1	37.2	37.7	38.5	37.2	37.8	
Proportion of women per practice (%)	53.8	53.9	53.5	53.3	53.2	53.0	52.9	53.2	52.1	52.5	<0.001
Proportion of men per practice (%)	46.2	46.1	46.5	46.7	46.8	47.0	47.1	46.8	47.9	47.5	

The data displayed in this table correspond to those of all patients followed in the general practices of interest (i.e., those with and without hypertension).

*p*-values were obtained using the Kruskal-Wallis test for the total number of patients and the mean age of patients per practice and the Chi-square test for the other variables.

### 3.2 Trends in the diagnosis of hypertension

**Figure 1:**
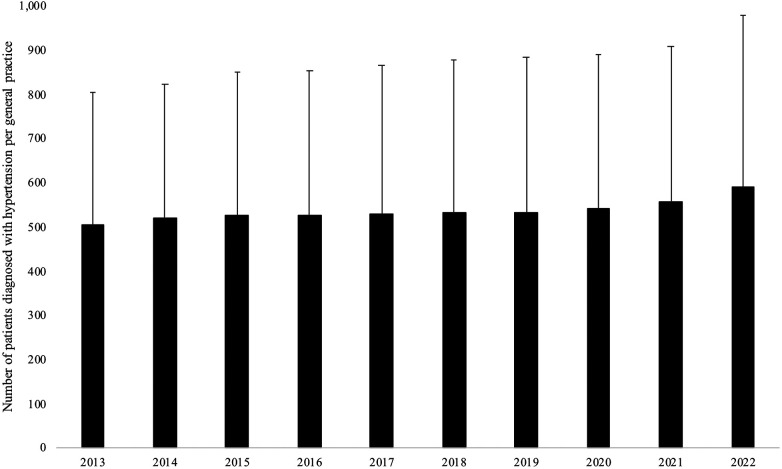
Trends in the number of patients diagnosed with hypertension per general practice in Germany between 2013 and 2022 in the overall population.

Figure 1 and Table 2 show the trends in the number of patients diagnosed with hypertension per general practice in Germany between 2013 and 2022 in the overall population and by age and sex. In the overall population, the unadjusted linear regression model revealed that the mean number of patients diagnosed with hypertension per practice significantly increased from 507 in 2013–592 in 2022 [beta coefficient (for an increase in one year) = 6.90, *p*-value < 0.001]. However, after adjusting for the total number of patients, the mean age, and the proportion of women per practice, there was a negative and significant relationship between years and the number of patients diagnosed with hypertension per practice (beta coefficient = −7.91, *p*-value < 0.001). The adjusted analyses conducted in the age and sex subgroups corroborated this finding, with the beta coefficient ranging from −0.27 in people aged 18–39 years to −4.13 in those aged ≥60 years. Similar results were obtained in the sensitivity analyses based on the data collected between 2013 and 2019 [unadjusted analysis (overall population): beta coefficient = 3.92, *p*-value = 0.141; adjusted analysis (overall population): beta coefficient = −7.58, *p*-value < 0.001].

**Table 2:** Trends in the number of patients diagnosed with hypertension per general practice in Germany between 2013 and 2022 in the overall population and by age and sex.

	2013	2014	2015	2016	2017	2018	2019	2020	2021	2022	Unadjusted analysis (2013–2022)^a^		Adjusted analysis (2013–2022)^a^		Unadjusted analysis (2013–2019)^a^		Adjusted analysis (2013–2019)^a^	
											Beta coefficient	*p*-value	Beta coefficient	*p*-value	Beta coefficient	*p*-value	Beta coefficient	*p*-value
By age																		
18–39 years	20 (16)	21 (15)	21 (17)	21 (16)	20 (16)	21 (16)	21 (17)	20 (16)	21 (17)	22 (19)	0.11	0.173	−0.27	<0.001	0.05	0.681	−0.16	0.081
40–59 years	145 (89)	147 (91)	148 (95)	146 (95)	145 (97)	143 (100)	141 (99)	141 (97)	144 (98)	148 (108)	−0.26	0.588	−3.51	<0.001	−0.83	0.284	−3.12	<0.001
≥60 years	341 (212)	352 (218)	359 (228)	361 (231)	365 (239)	370 (245)	371 (253)	382 (251)	393 (254)	422 (280)	7.06	<0.001	−4.13	<0.001	4.70	0.013	−4.29	0.002
By sex																		
Female	268 (160)	274 (164)	277 (171)	276 (172)	277 (179)	279 (185)	279 (188)	283 (185)	290 (187)	308 (207)	3.16	<0.001	−4.11	<0.001	1.57	0.269	−3.92	<0.001
Male	239 (143)	246 (146)	252 (154)	252 (156)	253 (161)	255 (164)	255 (168)	260 (166)	269 (167)	284 (185)	3.74	<0.001	−3.80	<0.001	2.35	0.065	−3.66	<0.001

Data are mean (standard deviation) unless otherwise specified.

COVID-19, coronavirus disease 2019.

^a^ The analyses corresponded to linear regression models with the years included as a continuous independent variable and the number of patients diagnosed with hypertension per practice as a continuous dependent variable. The linear regression models were unadjusted or adjusted for the total number of patients, the mean age, and the proportion of women per practice in the analysis based on the overall sample, the total number of patients and the proportion of women per practice in the age-stratified analyses, and the total number of patients and the mean age per practice in the sex-stratified analyses. The analyses were conducted for the periods 2013–2022 and 2013–2019 to investigate the effects of the COVID-19 pandemic on the trends.

### 3.3 Trends in the prescription of antihypertensive drugs

**Figure 2:**
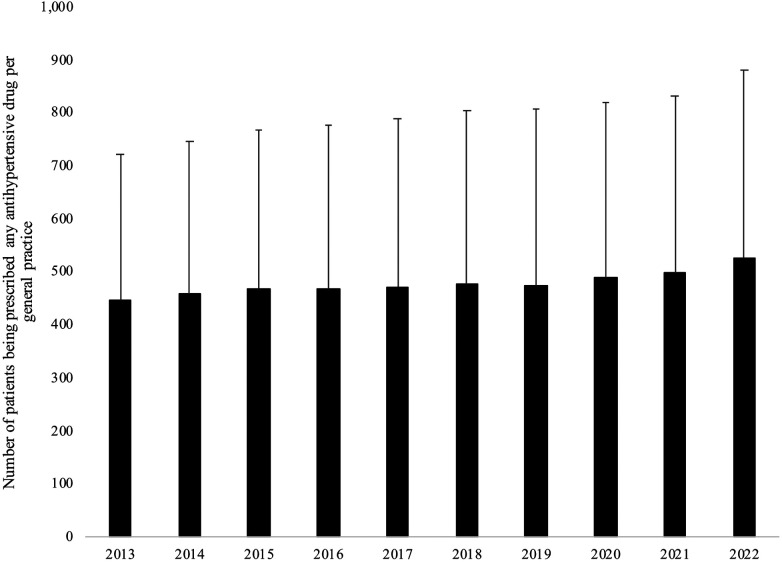
Trends in the number of patients being prescribed any antihypertensive drug per general practice in Germany between 2013 and 2022 in the overall population.

Figure 2 and Table 3 display the trends in the number of patients being prescribed antihypertensive drugs per general practice in Germany between 2013 and 2022 in the overall population. The number of individuals receiving any antihypertensive medication increased from 445 in 2013–527 in 2022 [beta coefficient (for an increase in one year) = 6.91, *p*-value < 0.001]. Nonetheless, the trends were decreasing after adjusting for the covariates mentioned above (beta coefficient = −5.40, *p*-value < 0.001). Similar negative and significant results were obtained for ACEis (beta coefficient = −7.02) and BBs (beta coefficient = −5.83). In contrast, non-significant associations were observed for CCBs (beta coefficient = 0.56) and diuretics (beta coefficient = −0.44), while there was a positive and significant association between years and ARBs (beta coefficient = 3.69). These findings were corroborated in the analyses which did not include data collected after 2019 [unadjusted analysis (any antihypertensive drug): beta coefficient = 4.63, *p*-value = 0.065; adjusted analysis (any antihypertensive drug): beta coefficient = −4.77, *p*-value = 0.012].

**Table 3:** Trends in the number of patients being prescribed different antihypertensive drugs per general practice in Germany between 2013 and 2022 in the overall population.

	2013	2014	2015	2016	2017	2018	2019	2020	2021	2022	Unadjusted analysis (2013–2022)^a^		Adjusted analysis (2013–2022)^a^		Unadjusted analysis (2013–2019)^a^		Adjusted analysis (2013–2019)^a^	
											Beta coefficient	*p*-value	Beta coefficient	*p*-value	Beta coefficient	*p*-value	Beta coefficient	*p*-value
ACEi	226 (143)	227 (145)	227 (150)	224 (150)	219 (150)	217 (151)	213 (153)	212 (149)	212 (147)	218 (152)	−1.70	0.016	−7.02	<0.001	−2.39	0.049	−6.65	<0.001
ARB	136 (100)	148 (107)	154 (113)	162 (122)	169 (129)	177 (139)	183 (145)	193 (147)	204 (154)	222 (168)	8.64	<0.001	3.69	<0.001	7.61	<0.001	4.09	<0.001
BB	242 (157)	247 (162)	249 (169)	248 (171)	246 (174)	247 (177)	243 (180)	247 (178)	248 (176)	259 (183)	0.81	0.324	−5.83	<0.001	−0.00	0.987	−5.26	<0.001
CCB	124 (85)	129 (87)	133 (92)	135 (96)	137 (100)	141 (104)	148 (113)	155 (115)	159 (117)	169 (122)	4.59	<0.001	0.56	0.156	3.50	<0.001	0.43	0.490
Diuretic	116 (81)	121 (83)	124 (87)	125 (89)	126 (91)	129 (96)	134 (105)	137 (107)	140 (107)	147 (111)	3.06	<0.001	−0.44	0.245	2.48	<0.001	−0.13	0.829

Data are mean (standard deviation) unless otherwise specified.

ACEi, angiotensin-converting enzyme inhibitor; ARB, angiotensin receptor blocker; BB, beta-blocker; CCB, calcium channel blocker; COVID-19, coronavirus disease 2019.

^a^ The analyses corresponded to linear regression models with the years included as a continuous independent variable and the number of patients being prescribed antihypertensive drugs per practice as a continuous dependent variable. The linear regression models were unadjusted or adjusted for the total number of patients, the mean age, and the proportion of women per practice. The analyses were conducted for the periods 2013–2022 and 2013–2019 to investigate the effects of the COVID-19 pandemic on the trends.

## 4 Discussion

### 4.1 Main findings

This study, which was conducted in 336 practices in Germany between 2013 and 2022 and included more than 750,000 patients per year, revealed that the number of those diagnosed with hypertension significantly decreased over time in the overall population. These findings were corroborated in the age- and sex-stratified analyses. In terms of the treatment of hypertension, the number of individuals receiving any antihypertensive drug significantly decreased between 2013 and 2022. Similar results were obtained in the analyses based on data exclusively collected prior to the COVID-19 pandemic. To the best of the knowledge of the authors, the present study is the first to investigate trends in the diagnosis of hypertension in the years following the COVID-19 pandemic and one of the first bodies of research analyzing trends in the prescription of antihypertensive drugs.

### 4.2 Interpretation of the findings

The decreasing trend in the number of adults diagnosed with hypertension observed in this research is in line with several prior studies. For example, in a sample of 38,276 individuals from the USA, there was a decrease in the age-standardized prevalence of hypertension from 48.4% in 1999–2000–45.4% in 2015–2016 (12). Another study, including 38,825 adults aged 30–79 years living in Norway, showed that the prevalence of hypertension decreased in all age (i.e., 30–39, 40–49, 50–59, 60–69, and 70–79 years) and sex groups (i.e., female and male) between 1979 and 2015 (17). A third analysis of 1,563 older adults (i.e., individuals aged ≥75 years) from Poland reported a decrease in the proportion of men with hypertension from 83.8% in 2007–77.9% in 2013–2014, the following figures being 75.4% and 71.8% in women (13). The present study adds to the literature by showing that decreasing trends in the diagnosis of hypertension persisted in the last years despite the human crisis related to the COVID-19 pandemic. Finally, it should also be noted that other studies have found either stable (10, 11, 14) or increasing trends in the prevalence of hypertension (9, 15, 16, 18). This discrepancy in the findings in the literature may be related to several factors, such as differences in the settings in which the studies of interest were conducted, differences in the years during which the data were collected, and differences in the definition of hypertension.

The decrease in the number of incident and prevalent cases of hypertension per year in general practices in Germany between 2013 and 2022 may reflect an improvement in the prevention of hypertension in the German population in recent years. This preliminary finding should be interpreted with caution pending future corroborating research. That being said, there is some evidence suggesting that risk factors for hypertension might have become less prevalent in Germany in the last decades. As a matter of fact, a study has investigated trends in health behaviors in this country between 1990 and 2011 (23). In this research, including 18,058 adults aged 25–69 years, a healthy lifestyle was defined as the presence of at least four out of five health behaviors (i.e., daily intake of both fruits and vegetables, sufficient physical activity, no current smoking, no current risk drinking, and body mass index between 18.5 and less than 25 kg/m^2^). It was observed that the prevalence of a healthy lifestyle increased from 9.3% in 1990–1992–13.5% in 1997–1999 and 14.7% in 2008–2011, with this increase being confirmed in most age and sex groups. It may be hypothesized that a healthy lifestyle has continued to become more frequent after 2011, potentially explaining, at least partially, the decrease in the number of patients diagnosed with hypertension in primary care each year in 2013–2022. Finally, the decrease in hypertension diagnosis in recent years could be related to some of the deleterious effects of the COVID-19 pandemic, such as decreased face-to-face consultations in general practices (24) and increased mortality in people with hypertension (25).

Another critical result of the study is the decrease in the number of adults being prescribed any antihypertensive drug per year between 2013 and 2022. This finding may be explained by the concomitant reduction in the number of patients diagnosed with hypertension each year. It should be noted that this study apart, little research has analyzed trends in the prescription of antihypertensive drugs. A survey of 53,496 adults living in the USA found that, among those with hypertension, there was an uptrend in the use of ACEis and ARBs and a downtrend in the use of BBs between 1999 and 2018 (18). Regarding CCBs, there was an increase in the use of these molecules until 2005 and a subsequent decrease after 2005. Opposite changes (i.e., decrease and then increase in the use) were observed for diuretics. These antihypertensive drug-specific trends differ from those reported in the present study, highlighting potential substantial differences in the pharmacological treatment of hypertension between the USA and Germany. These between-country differences aside, the increase in the number of patients being prescribed ARBs in the present research may be explained by the fact that, although the effects on blood pressure levels are comparable between ARBs and ACEis, ARBs display a better tolerability profile and are particularly effective in the prevention of major cardiovascular events (26). In this context, general practitioners may have favored the prescription of ARBs compared with other antihypertensive drugs over time.

### 4.3 Public health implications and directions for future research

Despite the fact that the study findings should be treated as preliminary, these results are reassuring and highlight a slight decrease in the number of people diagnosed with hypertension and treated for this chronic condition in general practices in Germany. Given that the decreasing trends are relatively modest, further public measures are warranted to better prevent the occurrence of hypertension in the general population. These measures may include, for example, education about the deleterious effects of alcohol on blood pressure levels, promotion of regular physical activity, and smoking cessation (27). In terms of future research, further studies are needed to corroborate or invalidate the present results in other settings and countries. Besides, more research is warranted on the differential trends by type of antihypertensive drug observed in this study.

### 4.4 Strengths and limitations

The major strengths of the study are the large sample size, the use of data collected until 2022, and the use of diagnosis and prescription data relying on the ICD-10 and EphMRA classification, respectively. Nonetheless, the study findings should be interpreted in the light of several limitations. First, there is no information on blood pressure, although blood pressure data would have allowed more detailed analyses. More specifically, it was not known if the diagnosis of hypertension relied on a sphygmomanometer or 24 h ambulatory blood pressure monitoring. Second, there are no data in the Disease Analyzer database on health behaviors (e.g., alcohol consumption and smoking status), and it was, therefore, not possible to adjust the analyses for these factors. Third, this study only included general practices, and the results cannot be extrapolated to specialized practices or hospital settings.

### 4.5 Conclusion

Overall, this study found decreasing trends in the number of patients diagnosed with and treated for hypertension in general practices in Germany between 2013 and 2022. Although these findings should be interpreted with caution, they might indicate that the prevention of hypertension has improved in German general practices in recent years. Finally, more studies are warranted to corroborate or invalidate these results in other settings in Germany and other countries.

## Data Availability

The data used for the present study are available from the corresponding author upon reasonable request. Requests to access these datasets should be directed to Sarah Krieg; sarah.krieg@mara.de.
