# Supplementary material for: Trends in hypertension and hypertension treatment in primary care in general practices in Germany between 2013 and 2022
Source: Front Cardiovasc Med. 2024 Jun 12;11:1390902. doi: 10.3389/fcvm.2024.1390902 (PMC11204120; doi:10.3389/fcvm.2024.1390902)
Supplement: Supplementary file 1 [file Datasheet1.pdf]

Trends in hypertension and hypertension treatment in primary care in Germany between 2013 and 2022

Background

Mixed findings on hypertension trends

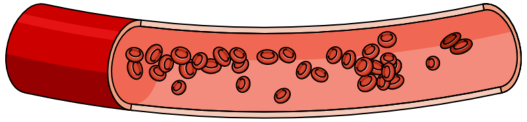

Critical need for more research on the topic

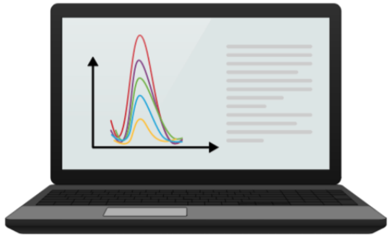

Methods

Retrospective cohort study

336 general practices from Germany (IQVIA)

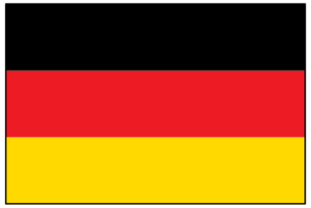

Diagnoses and prescriptions based on the ICD-10 and EphMRA classification, respectively

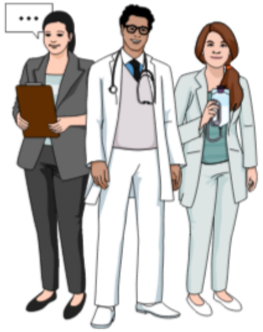

Findings

Around 2,200-2,800 patients per practice per year

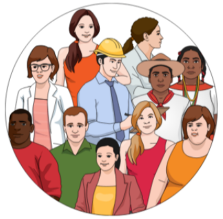

Decrease in the number of patients being diagnosed or being treated for hypertension

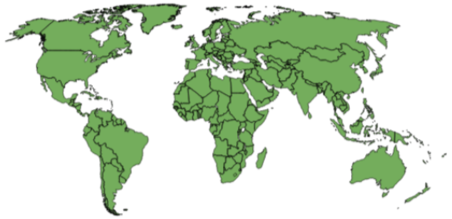

These data need to be corroborated
